# Epac1 Blocks NLRP3 Inflammasome to Reduce IL-1*β* in Retinal Endothelial Cells and Mouse Retinal Vasculature

**DOI:** 10.1155/2017/2860956

**Published:** 2017-02-28

**Authors:** Youde Jiang, Li Liu, Elizabeth Curtiss, Jena J. Steinle

**Affiliations:** ^1^Department of Anatomy and Cell Biology, Wayne State University School of Medicine, Detroit, MI, USA; ^2^Department of Ophthalmology, Wayne State University School of Medicine, Detroit, MI, USA

## Abstract

Inflammation is an important component of diabetic retinal damage. We previously reported that a novel *β*-adrenergic receptor agonist, Compound 49b, reduced Toll-like receptor 4 (TLR4) signaling in retinal endothelial cells (REC) grown in high glucose. Others reported that TLR4 activates high-mobility group box 1 (HMGB1), which has been associated with the NOD-like receptor 3 (NLRP3) inflammasome. Thus, we hypothesized that Epac1, a downstream mediator of *β*-adrenergic receptors, would block TLR4/HMGB1-mediated stimulation of the NLRP3 inflammasome, leading to reduced cleavage of caspase-1 and interleukin-1 beta (IL-1*β*). We generated vascular specific conditional knockout mice for Epac1 and used REC grown in normal and high glucose treated with an Epac1 agonist and/or NLRP3 siRNA. Protein analyses were done for Epac1, TLR4, HMGB1, NLRP3, cleaved caspase-1, and IL-1*β*. Loss of Epac1 in the mouse retinal vasculature significantly increased all of the inflammatory proteins. Epac1 effectively reduced high glucose-induced increases in TLR4, HMGB1, cleaved caspase-1, and IL-1*β* in REC. Taken together, the data suggest that Epac1 reduces formation of the NLRP3 inflammasome to reduce inflammatory responses in the retinal vasculature.

## 1. Introduction

The role of inflammation and inflammatory mediators in the diabetic retina is becoming increasingly crucial for the development of pathology [1, 2]. While a large number of potential inflammatory mediators are found in the diabetic rodent and human retina [3–5], the mechanisms to inhibit activation of these factors have proven more difficult to elucidate. Recent evidence has suggested that the diabetic environment (particularly type 2 diabetes) may provide the appropriate triggers to activate inflammasomes, specifically the NOD-like receptor 3 (NLRP3) inflammasome [6, 7]. Inflammasomes are expressed in the cytosol and form in response to microbial or danger signals [8]. Activation of the inflammasome leads to the cleavage of caspase-1 and activates interleukin-1 beta (IL-1*β*) [8, 9]. A number of factors can lead to the activation of an inflammasome, including reactive oxygen species, endoplasmic reticulum stress, and cationic fluxes [9]. Since most of these same factors are observed in the diabetic retina, it is probable that inflammasomes may contribute to diabetic retinal pathology. Reports have shown that acute glaucoma is associated with increased high-mobility group box 1 (HMGB1) leading to activation of the NLRP3 inflammasome [10]. Similarly, work in age-related macular degeneration has suggested that NLRP3 is upregulated in the retinal pigmented epithelial (RPE) cells [11]. Focusing on the diabetic retina, one study investigated RPE cells cultured in high glucose and showed an increase in NLRP3 inflammasome activation and IL-1*β* secretion in these cells [12]. However, RPE cells are not a predominant cell type affected in the diabetic retina. We have focused our work on retinal endothelial cells, as these cells are significantly affected in the diabetic retinal milieu.

We recently reported that a novel *β*-adrenergic receptor agonist, Compound 49b, could reduce Toll-like receptor 4 (TLR4) signaling in retinal endothelial cells (REC) grown in high glucose [13], suggesting that *β*-adrenergic receptors could regulate innate immune responses in the retina. *β*-Adrenergic receptors can signal through cAMP to activate protein kinase A (PKA) or exchange protein activated by cAMP (Epac). Both Epac1 and Epac2 have been localized in the retina [14], and we recently reported that Epac1 can regulate IL-1*β* and inflammatory mediators in the retina [15]. Others have also suggested that Epac1 can regulate inflammation in murine macrophages [16]. Similarly, work in Raw 264.7 macrophages showed that Epac1 can regulate TLR4 [17].

Thus, in this study, we wanted to investigate whether Epac1 regulated the NLRP3 inflammasome to reduce IL-1*β* release in the retina. For this work, we used primary human retinal endothelial cells in culture, as well as vascular specific Epac1 conditional knockout mice.

## 2. Materials and Methods

### 2.1. Vascular Specific Epac1 Knockout Mice

All animal procedures met the Association for Research in Vision and Ophthalmology requirements and were approved by the Institutional Animal Care and Use Committee of Wayne State University and conformed to NIH guidelines. Epac1 floxed mice (B6; 129S2-Rapgef3^tm1Geno/J^ mice) and B6 FVB-Tg (cdh5-cre)7Mlia/J Cre mice were purchased from Jackson Laboratories. After 2 generations, the Epac1 floxed mice were bred with cdh5-Cre mice to generate conditional knockout mice in which Epac1 is eliminated in vascular endothelial cells [15]. At 3 months of age, Epac1 floxed and Epac1 Cre-Lox mice were used for experiments.

### 2.2. Retinal Endothelial Cells (REC)

Primary human retinal endothelial cells (REC) were acquired from Cell Systems Corporation (CSC, Kirkland, Washington). Cells were grown in Cell Systems medium supplemented with microvascular growth factors (MVGS), 10 ug/mL gentamycin, and 0.25 ug/mL amphotericin B (Invitrogen, Carlsbad, CA) on attachment factor coated dishes. Once cells reached confluence, some dishes were moved to Cell Systems medium supplemented with D-glucose at 25 mM. Only cells prior to passage 6 were used. Cells were quiesced by incubating in high or normal glucose medium without MVGS for 24 h prior to experimental use.

### 2.3. Treatments

REC in high glucose (25 mM) were transfected with NLRP3 siRNA (SMARTpool: ON-TARGETplus RAPGEF3 siRNA, Dharmacon, Lafayette, CO) or scrambled siRNA at a final concentration of 20 nM using RNAiMAX transfection reagent according to the manufacturer's instructions. Twenty-four hours after transfection, some cells were treated with an Epac1 agonist (8-CPT-2′-O-Me-cAMP) for an additional 2 hours at 10 uM. Analyses were done on REC samples after 3-4 days in normal or high glucose and 2 hours after Epac1 stimulation.

### 2.4. Cytoplasmic Isolation for HMGB1

Some retinal lysates from the mice and REC were immediately processed for nuclear versus cytosolic extraction using the NE-PER kit (Thermo Scientific, Pittsburgh, PA). For these samples, extraction procedures were done according to the manufacturer's instructions. After extraction of each cellular portion, western blotting was done on only the cytoplasmic fraction.

### 2.5. Western Blotting

Whole retinal lysates from mice or cell culture lysates were collected in a lysis buffer containing protease and phosphatase inhibitors. Equal amounts of protein were separated onto a precast Tris-glycine gel (Invitrogen, Carlsbad, CA) and blotted onto nitrocellulose membrane. After blocking in TBST (10 mM Tris-HCl buffer, pH 8.0, 150 mM NaCl, and 0.1% Tween 20) and 5% (w/v) BSA, the membranes were treated with Epac1, TLR4, NLRP3, HMGB1, cleaved caspase-1 (Abcam, Cambridge, MA), or beta-actin (Santa Cruz Biotechnology, Santa Cruz, CA) primary antibodies followed by incubation with secondary antibodies labeled with horseradish peroxidase. Antigen-antibody complexes were detected by chemiluminescence reagent kit (Thermo Scientific, Pittsburgh, PA) and data was acquired using an Azure C500 (Azure Biosystems, Dublin, CA). Western blot data were assessed using Image Studio Lite software.

### 2.6. ELISA

IL-1*β* ELISA was completed according to the manufacturer's instructions with the exception of 120 ug protein loaded into all wells, with the primary antibody incubated overnight.

### 2.7. Statistical Analyses

Nonparametric Kruskal-Wallis tests with Dunn's post hoc test were used for the cell culture data. One-way ANOVA with Student's Newman-Keuls post hoc test was used for animal work. *P* < 0.05 was considered statistically significant.

## 3. Results

### 3.1. Loss of Epac1 in Endothelial Cells Increased Retinal Levels of TLR4 and Cytoplasmic HMGB1

**Figure 1:**
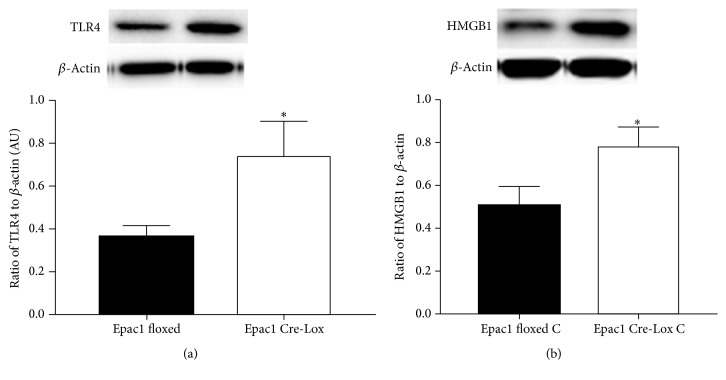
Loss of Epac1 increased TLR4 and cytosolic HMGB1. Western blot data from retinal lysates from Epac1 floxed and Epac1 Cre-Lox mice for TLR4 (a) and cytosolic HMGB1 (b). ^*∗*^*P* < 0.05 versus Epac1 floxed. *N* = 6. Data are mean ± SEM.

Since we had reported that Compound 49b could reduce TLR4 signaling [13], we wanted to determine whether this was through Epac1 actions. We measured protein levels of TLR4 in retinal lysates from Epac1 floxed or Epac1 conditional knockout mice and found that loss of Epac1 significantly increased TLR4 levels in the retina (Figure 1(a)) compared to the Epac1 floxed mice. Since TLR4 often signals through HMGB1 to mediate inflammation [18, 19], we also measured cytosolic levels of HMGB1 in the mice. Loss of Epac1 significantly increased cytosolic levels of HMGB1, suggesting that Epac1 may be key to anti-inflammatory actions of Compound 49b.

### 3.2. NLRP3 and Cleaved Caspase-1 Are Increased in the Retina of Conditional Epac1 Knockout Mice

**Figure 2:**
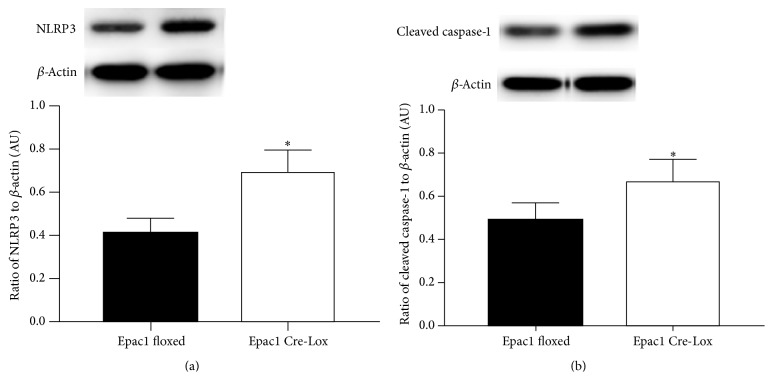
Epac1 reduces formation of the NLRP3 inflammasome and the cleavage of caspase-1. Western blot data from retinal lysates from Epac1 floxed and Epac1 Cre-Lox mice for NLRP3 (a) and cleaved caspase-1 (b). ^*∗*^*P* < 0.05 versus Epac1 floxed. *N* = 6. Data are mean ± SEM.

Others have reported that HMGB1 can be associated with NLRP3 inflammasomes in acute glaucoma [10]. Since Epac1 reduced cytosolic HMGB1 levels, we wanted to determine whether Epac1 also could modulate NLRP3. We found that loss of Epac1 in mouse retinal vasculature led to increased NLRP3 (Figure 2(a)) and cleaved caspase-1 (Figure 2(b)). Since activation of NLRP3 can activate cleaved caspase-1 to increase IL-1*β* to mediate inflammation, our data showing increased NLRP3 and cleaved caspase-1 in Epac1 conditional knockout mice suggest that Epac1 may regulate these actions. We have previously reported that Epac1 Cre-Lox mice have increased IL-1*β* [15].

### 3.3. An Epac1 Agonist Can Significantly Reduce TLR4 and Cytoplasmic HMGB1 Levels in High Glucose Treated REC

**Figure 3:**
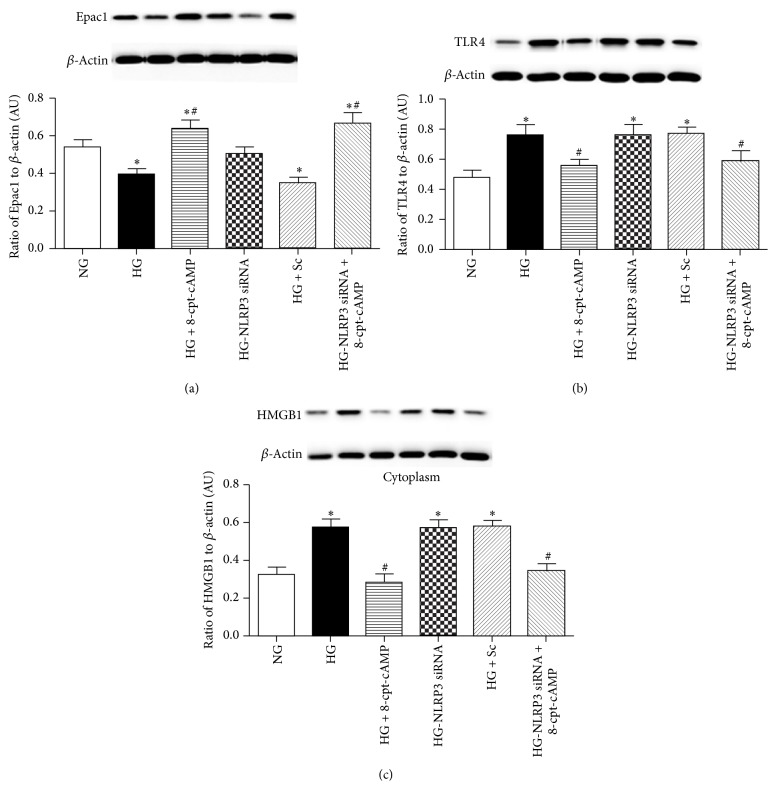
NLRP3 siRNA does not affect TLR4 and HMGB1 levels after Epac1 agonist treatment. Western blot data from REC grown in normal glucose (NG), high glucose (HG), high glucose + Epac1 agonist (HG + 8-cpt-cAMP), high glucose + NLPR3 siRNA (HG + NLPR3 siRNA), high glucose + scrambled siRNA (HG + Sc), and high glucose + Epac1 +NLRP3 siRNA (HG + NLRP3 siRNA + 8-cpt-cAMP) for Epac1 (a), TLR4 (b), and cytosolic HMGB1 (c). *N* = 6. Data are mean ±SEM. ^*∗*^*P* < 0.05 versus NG; ^#^*P* < 0.05 versus HG.

To support our work in the conditional knockout mice, we also grew REC in normal and high glucose and measured TLR4 and HMGB1 levels after treatment with an Epac1 agonist.  Figure 3(a) shows that high glucose significantly reduced Epac1 levels, which were restored following Epac1 agonist treatment. The Epac1 agonist also significantly reduced high glucose-induced increases in TLR4 (Figure 3(b)) and cytosolic HMGB1 (Figure 3(c)) compared to high glucose only. Use of the NLRP3 siRNA did not affect these levels, as it is likely that TLR4 and HMGB1 are upstream of the activation of the NLRP3 inflammasome [7, 8].

### 3.4. Epac1 and NLRP3 Can Reduce Cleaved Caspase-1 and IL-1*β* Levels in REC Cultured in High Glucose

**Figure 4:**
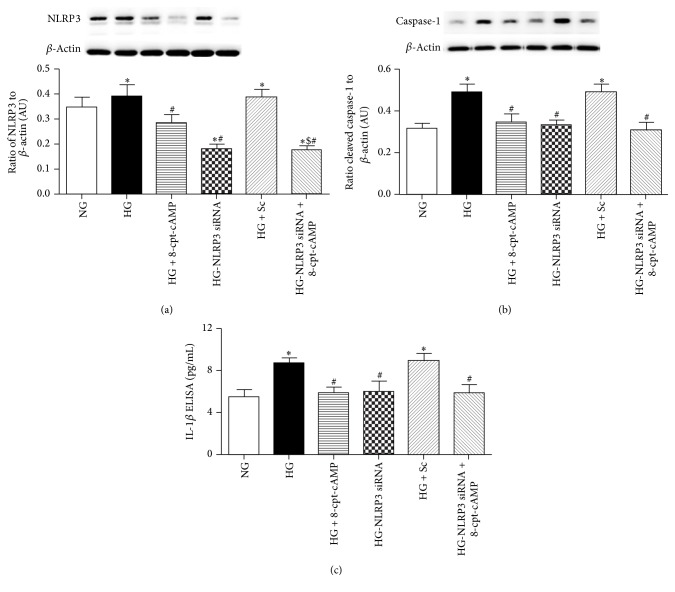
Epac1 and NLRP3 both can reduce cleavage of caspase-1 and IL-1*β*. Protein analyses from REC grown in normal glucose (NG), high glucose (HG), high glucose + Epac1 agonist (HG + 8-cpt-cAMP), high glucose + NLPR3 siRNA (HG + NLRP3 siRNA), high glucose + scrambled siRNA (HG + Sc), and high glucose + Epac1 agonist + NLRP3 siRNA (HG + NLRP3 siRNA + 8-cpt-cAMP) for NLRP3 (a), cleaved caspase-1 (b), and IL-1*β* (c). *N* = 6 for NLRP3 and cleaved caspase-1; *N* = 5 for ELISA for IL-1*β*. Data are mean ± SEM. ^*∗*^*P* < 0.05 versus NG; ^#^*P* < 0.05 versus HG; ^$^*P* < 0.05 versus HG+8-cpt-cAMP.

REC grown in high glucose have increased levels of NLRP3, cleaved caspase-1, and IL-1*β*, which is reduced by treatment with an Epac1 agonist (Figures 4(a)–4(c)). These data agree with the data from mouse retinal lysates. Additionally, NLPR3 siRNA also significantly reduced cleaved caspase-1 (Figure 4(b)) and IL-1*β* (Figure 4(c)) levels in REC. When the Epac1 agonist and NLP3 siRNA were combined, the response was similar to the Epac1 agonist or NLPR3 siRNA alone. Taken together with the mouse data, Epac1 reduces NLRP3 levels, leading to reduced inflammation mediated by cleaved caspase-1 and IL-1*β*.

## 4. Discussion

Few would argue that inflammation is a key component of the retinal damage associated with diabetes. While inhibition of specific inflammatory mediators has been effective in resolving some of the complications in rodents [4, 20, 21], increased understanding of upstream pathways may allow for more specific treatments or broad spectrum inhibition of the inflammatory process. Inhibition of receptor for advanced glycation end products (RAGE) has also proven effective in a rodent model of diabetes [22, 23]; however, other targets of TLR4 may also be effective. TLR4 can activate cytosolic HMGB1, leading to the initiation of inflammation [19, 24]. In acute glaucoma, HMGB1 activates the NLRP3 inflammasome, thus triggering the immune response [10]. We have previously reported that TLR4 signaling is increased in REC grown in high glucose, which could be inhibited by Compound 49b [13]. In this work, we expanded those cell culture studies to demonstrate that Epac1, a downstream mediator of Compound 49b actions, is key to the reduction in TLR4 levels. Loss of Epac1 in the mouse retinal vasculature also led to a significant increase in cytosolic HMGB1.

Because others have suggested that danger associated molecular patterns (DAMPs) can activate inflammasomes in type 2 diabetes [7], we wanted to investigate whether the cytosolic HMGB1 could stimulate formation of the NLRP3 inflammasome in the retina, as well as the role of Epac1 in this activation. Data demonstrate that Epac1 can inhibit NLRP3 levels, as well as the cleavage of caspase-1 in the retinal vasculature. We have already reported that loss of Epac1 increased IL-1*β* levels in the retina [15]. While there is little literature on Epac1 regulation of the NLRP3 inflammasome, there is work to support an anti-inflammatory role of Epac1 in macrophages [16, 17].

**Figure 5:**
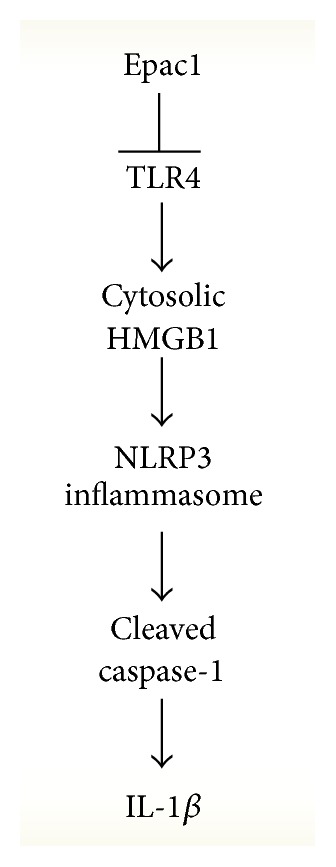
Schematic of the proposed regulation of the NLRP3 inflammasome by Epac1.

To support our mouse retinal data, we also grew primary REC in normal and high glucose and found that an Epac1 agonist could significantly reduce both TLR4 and cytosolic HMGB1, which was not altered by NLRP3 siRNA, suggesting that both TLR4 and HMGB1 are upstream of NLRP3. However, cleaved caspase-1 and IL-1*β* levels were not reduced by an Epac1 agonist when cells were transfected with NLRP3 siRNA. Taken together, these data suggest that Epac1 reduces TLR4 and HMGB1 to inhibit the NLRP3 inflammasome activation in the retinal vasculature (Figure 5). In future work, these studies will be expanded into the diabetic retina in vivo. Additionally, it is highly likely that other factors can also regulate the NLRP3 inflammasome in the diabetic retina, including thioredoxin interacting proteins (TXNIP) [6]. Interestingly, both NLRP3 and TXNIP knockout mice have reduced insulin resistance [25]. Thus, it will be interesting to investigate the role of Epac1 in these pathways.

## 5. Conclusions

These studies demonstrate that loss of Epac1 can increase inflammatory signaling in the retina, specifically TLR4/HMGB1-mediated activation of the NLRP3 inflammasome and IL-1*β* secretion. TLR4 and HMGB1 are upstream of NLRP3 as they were unaffected by siRNA treatment in Epac1-stimulated REC grown in high glucose. Mouse retinal samples showed increased inflammatory pathways in vascular specific Epac1 conditional knockout mice. Epac1 may serve as a novel broad spectrum anti-inflammatory factor for the retina through inhibition of the NLRP3 inflammasome.
